# Reduced Use of Antibiotics and Nasal Decongestants During Treatment with a Mouthwash Containing Delmopinol

**DOI:** 10.3290/j.ohpd.b4586769

**Published:** 2023-11-02

**Authors:** Torgny Sjödin, Rolf Movert, Mikael Åström

**Affiliations:** a R&D Director, Biosurface AB; Visiting Scientist, Faculty of Odontology, Malmö University, Malmö, Sweden (retired). Main author, interpreted the results.; b Director, Clinical Department , Biosurface AB, Malmö, Sweden (retired). Study design, wrote the manuscript.; c StatCons, Malmö, Sweden. Statistical evaluation, wrote the manuscript.

**Keywords:** antibiotics, Decapinol, decongestants, delmopinol, reduction

## Abstract

**Purpose:**

To evaluate the use of concomitant medication in combination with a mouthwash of delmopinol HCl 0.2% based on data from 8 phase III efficacy studies on the mouthwash.

**Materials and Methods:**

Clinical data obtained from 8 previously performed phase III studies, carried out to document the clinical efficacy of a mouthwash of delmopinol HCl 0.2% with respect plaque and gingivitis, were used to analyse the use of concomitant medication. In these 8 randomised double-blind clinical phase III studies the patients were – in addition to their normal oral hygiene measures – treated for 2–6 months with mouthwashes containing delmopinol HCl 0.2%, delmopinol HCl 0.1%, chlorhexidine digluconate 0.2% or placebo. The number of visits in each study was three. Each time the patients visited the dentist for efficacy determinations, other data were also recorded. One of these was whether the patient had used any other medication during the study. In this paper, the number of treatments of different types of concomitant medication (antibiotics, nasal decongestants and others) was used as a basis for statistical comparisons between the different test groups.

**Results:**

For antibiotics (all indications), a 27% lower number of treatments was obtained in the delmopinol 0.2% group in comparison with the placebo group, and a 41% decrease was observed for treatments with antibiotics for respiratory infections. For nasal decongestants, the number of treatments was 53% lower in the delmopinol 0.2% group.

**Conclusions:**

The delmopinol HCl 0.2% solution in patients with gingivitis provided a statistically significant reduction of concomitant use of antibiotics and nasal decongestants.

It is well recognised that imbalances in the resident oral microflora lead to dental diseases such as caries and periodontal diseases. During the last decades, much attention has been focused on the systemic consequences of these oral infections as risk factors for other diseases. Especially periodontitis has been related to several systemic conditions, including respiratory disorders, adverse pregnancy outcomes, cardiovascular diseases, type 2 diabetes mellitus, chronic renal disease and metabolic syndrome, a connection that is considered to depend on the low inflammatory burden associated with periodontitis.^14^ The associations between dental caries and systemic health, however, have received little attention.^30^

By being an important interface between the body and the external environment, the mouth is an entry for possible microbiological pathogens from food and air. Improved oral hygiene is therefore beneficial, not only in order to reduce dental diseases, but also for the general health of an individual. It has therefore been suggested that oral disinfectants may be a valuable tool, especially for subjects who are at risk (e.g., people in intensive care, immunocompromised people, elderly, patients with cystic fibrosis) and where oral hygiene is poor.^9,20^

Delmopinol is a tertiary amine surfactant, developed as an anti-plaque agent to reduce plaque and gingivitis as an adjunct to normal mechanical cleaning where this has proved inadequate. The surface-active compound has relatively low antimicrobial properties but promotes a microbial flora compatible with dental health.^11,32^ It binds to hard and soft oral tissues as well as to bacterial surfaces and affects several of the steps in the formation and establishment of dental biofilms.^11,21,22,26,28,29,31,32,38,39^ Clinical phase II studies have shown that a shift exists in the quality of plaque towards a microflora associated with gingival health and demonstrated that the compound is effective in reducing the level of gingivitis.^6,7,27^ Furthermore, a clinical study has shown delmopinol to dissolve established plaque.^17^ Studies in humans and rats showed that delmopinol is rapidly adsorbed and retained in the oral mucosa and then slowly diffuses out of this tissue into the systemic circulation.^12,13,34,35^

The clinical phase III studies on Decapinol Mouthwash (at the time of the clinical studies: Pharmacia AB; Uppsala, Sweden) 2 mg/ml formulation of delmopinol (subsequently called delmopinol 0.2%) in patients with gingivitis, with the included eight double-blind, placebo controlled studies with a treatment time of 2-6 months, showed that rinsing with delmopinol hydrochloride 0.2% fulfilled ADA effectiveness criteria for controlling plaque and gingivitis.^5,10,15,16,18^ The efficacy and safety results are summarised in a meta-analysis study by Addy et al.^1^ In these studies, the effect and adverse event profile of delmopinol hydrochloride 0.2% was recorded each time the patients visited the institution in a Case Record Form (CRF). However, also other data, e.g., possible use of concomitant medication, were noted in the CRFs.

The completion of this programme of studies produced a substantial data bank, based on which we recently published a paper^36^ reporting a significantly lower frequency of aphthous stomatitis compared to placebo. However, there were also clear indications that upper respiratory tract infections were less frequent among patients using the formulation with delmopinol 0.2% in comparison with the placebo-treated patients. This positive finding prompted interest in investigating whether rinsing with delmopinol 0.2% also had an effect on the use of concomitant medication.

The purpose of this work was thus to perform a statistical evaluation of clinical data from the 8 phase III studies on a mouthwash formulation of delmopinol HCl 0.2% regarding the patient’s use of concomitant medication. Of special interest were drugs such as antibiotics and others commonly used in connection with respiratory diseases and the common cold.

## Materials and Methods

### Study Design

The concomitant medication data used in the present paper were obtained from 8 clinical phase III trials previously performed to investigate the effect on gingivitis and plaque of a mouthwash formulation of delmopinol HCl 2 mg/ml. Since these clinical trials thus are essential as a data resource for the calculations carried out in this study, their design is outlined below:

All studies were randomised and double-blind, with a parallel-group design, where five studies involved supervised rinsing and three involved unsupervised rinsing. In the supervised studies, all weekday mouthrinsing was observed by the staff. Weekend rinsing was not supervised, but the patients kept a record of each rinsing. The length of the studies was 2–6 months. The studies were conducted by independent university-based research groups of high academic standing in the field of oral hygiene research (Table 1) and in accordance with the principles of good clinical practice.

**Table 1:** Number of patients and length of treatment in the phase III programme

Study Number	Number of patients entering treatment				Number (and percent) of patients completing treatment				Study length	Study site
	Placebo	Delmop. 0.1%	Delmop. 0.2%	Chlorhexidine	Placebo	Delmop. 0.1%	Delmop. 0.2%	Chlorhexidine		
DEC-89016*	40	40	40	-	40(100)	40(100)	40(100)	-	8 weeks	Dept. of Periodontol, Inst. of Postgraduate Dental Education, Jönköping, Sweden
DEC-89017*	39	44	38	-	39(100)	44(100)	38(100)	-	8 weeks	Dept. of Periodontology, University of Umeå, Sweden
DEC-90014*	53	-	53	50	51(96)	-	50(94)	41(82)	6 months	Dept. of Periodontology, University of Bern, Switzerland
DEC-90018*	46	-	49	48	43(93)	-	48(98)	42(88)	6 months	Dept. of Periodontology, Lund University, Malmö, Sweden
DEC-90019	157	157	156	-	157(100)	157(100)	156(100)	-	3 months	Dept. of Periodontology, University of Dublin, Ireland
DEC-90023*	51	-	50	52	47(92)	-	47(94)	47(90)	5 months	School of Dental Medicine, University of Brussels, Belgium
DEC-91025	150	150	150	-	147(98)	147(98)	142(95)	-	6 months	University Hospital of Wales, Cardiff
DEC-91027	150	151	150	-	147(99)	148(99)	146(97)	-	3 months	Dept. of Periodontology, Faculty of Medicine, Catholic University, Leuven, Belgium
Total number	686	542	686	150	671(98)	536(99)	667(97)	130(87)		

* The asterisk indicates that the study was supervised.

In all studies, patients were asked to continue with their habitual oral hygiene measures and to use their usual toothpaste. In addition, the patients were instructed to rinse twice daily (morning and evening) for 60 s with 10 ml Decapinol Mouthwash 2 mg/ml (containing delmopinol HCl 2 mg/ml) or placebo (the vehicle of Decapinol Mouthwash).^1^ In some studies, chlorhexidine digluconate 2 mg/ml (Hibitane Dental 0.2%, subsequently called chlorhexidine 0.2%) or Decapinol Mouthwash 1 mg/ml (delmopinol 0.1%) were used as active controls (see Table 1). In addition to the active substance, the formulations of Decapinol Mouthwash contained flavouring and sweetening agents as well as a low (1.5%) concentration of ethanol. The solutions of delmopinol and placebo were unbuffered and their pH was adjusted to 5.5. Packaging and labeling of the solutions were carried out at independent clinical service departments and were dispensed in identical amber glass bottles.

The patients included in the studies had mild to severe gingivitis, with a frequency of bleeding on probing from about 25% to more than 95% at baseline. The range of age was from 18 to 73 years, with about 10% of the patients older than 40 years. Fifty percent of the patients had at least one pocket ≥4 mm deep, and 22% pockets ≥ 5 mm or deeper. The deepest pocket was 10 mm. Patients with more than four pockets >5 mm deep were excluded from the studies. Both sexes were included in the studies, except in studies DEC-89016, DEC-89017 and DEC-90018, where all patients were men. Overall, about 60% of the included patients were men and 40% were women. The major part of the patients (61%) were non-smokers and 20% were regular smokers. The remaining 19% smoked “sometimes”. Patients were allocated to treatment groups according to a computer-generated randomisation list. All details regarding inclusion and exclusion criteria are summarised in Table 2.

**Table 2:** Summary details of the participation criteria for the patients in all eight studies

Inclusion conditions	Exclusion conditions
Age ≥ 18 years Minimum of 16 natural teeth without crowns, bridgework or defective dental restorations Gingivitis, defined as bleeding on probing at ≥ 25% of six sites around each tooth (all unsupervised studies and three supervised studies) or gingival Silness-Löe index score ≥ 2 at ≥ 25% of sites (two supervised studies) Written informed consent Women of child-bearing potential fully informed of the toxicological status of delmopinol and adequately equipped with contraceptives	Removable partial dentures Caries with cavities More than four pockets deeper than 5 mm (excluding distal site of second molar and all third molar sites) Known hypersensitivity to any study treatment Drug or alcohol addiction Severe liver or kidney disease, or severely ill patients with multiple drug requirements Antibiotic treatment within immediately preceding 6 weeks Psychiatric disorders Current and ongoing use of anti-inflammatory or anticholinergic drugs Pregnancy or pregnancy planned Breast feeding

Sub- and supragingival professional cleaning was administered to all patients in every study after the baseline assessments had been completed, and they were instructed to continue with habitual oral hygiene measures and use their usual toothpaste. Any rinsing undertaken in conjunction with toothbrushing was to be performed after mechanical cleaning. Efficacy outcomes comprised the modified plaque index, modified gingival index (MGI) and gingival bleeding on probing (BOP). Special attention was paid to the blindness, and an independent observer was appointed in each study to handle questions on adverse events as well as their observation and recording. Thus, one investigator measured the efficacy variables and another person dealt with the adverse events. Both investigators were unaware of the determinations made by the other investigator and used separate case record forms (CRF).

Each time the patients visited the institution for efficacy determinations, additional data – e.g., adverse events and concomitant medication – were recorded. There were three visits in each study (baseline, mid, final), except in one of the studies (DEC-91023) with four visits (baseline, 6 weeks, 3 months, 5 months).

The protocols of all the studies were approved by local ethics committees and the trials themselves were conducted in accordance with the provisions and principles of the World Medical Assembly Declaration of Helsinki (1964 and later amendments) and good clinical practice.

### Statistics

The total number of treatment periods for different types of concomitant medication (antibiotics, nasal decongestants and others) formed the basis for the calculations and were compared with the number of treatment periods in the different test groups (delmopinol 0.2%, delmopinol 0.1%, chlorhexidine 0.2% and placebo). Fisher’s exact test was used for statistical analysis.

When calculating the percentage difference of concomitant medication in the delmopinol group in comparison to placebo the following formula was used:


$$\frac{(\text{Fraction in placebo group}-\text{fraction in delmopinol group})\times 100}{\text{Fraction in placebo group}}$$

Delmopinol 0.2% and placebo solutions were used in all phase III studies, while the delmopinol 0.1% was used in five, and chlorhexidine 0.2% in three of the eight studies.

The statistical significance tests were performed on pooled data of the following three sets of studies:

- Set 1: Delmopinol 0.2% compared to placebo: all of the phase III studies.
- Set 2: Delmopinol 0.2% compared to delmopinol 0.1% and placebo: study Nos. DEC-89016, DEC-89017, DEC-90019, DEC-91025 and DEC-91027.
- Set 3: Delmopinol 0.2% compared to chlorhexidine 0.2% and placebo: study Nos. DEC-90014, DEC-90018 and DEC-90023.

Statistical significance was set at p < 0.05.

## Results

The number of patients and length of treatment in the phase III programme are shown in Table 1, which also shows which studies were supervised and unsupervised. The number of patients entering the different sets of studies is shown in Table 3.

**Table 3:** Number of patients entering in different sets of studies (baseline, ITT)

Set No.	Placebo	Delmopinol 0.1%	Delmopinol 0.2%	Chlorhexidine 0.2%
1	686	-	686	-
2	536	542	534	-
3	150	-	152	150

Set 1: includes placebo and delmopinol 0.2% in all phase III studies; Set 2: includes placebo, delmopinol 0.1% and delmopinol 0.2% in study Nos. DEC-89016, DEC-89017, DEC-90019, DEC-91025 and DEC-91027; Set 3: includes placebo, delmopinol 0.2% and chlorhexidine 0.2% in study Nos. DEC-90014, DEC-90018 and DEC-90023.

### Antibiotics (Group J01)

The number of antibiotic treatments and the number of treated patients in the delmopinol 0.2% and the placebo groups are shown in Table 4.

**Table 4:** Number of antibiotic treatments and the number of patients treated in the delmopinol 0.2% and placebo groups, per study and in total

Study number	Delmopinol 0.2%		Placebo	
	Number of treatments	Number of patients treated	Number of treatments	Number of patients treated
DEC-89016	2	2	1	1
DEC-89017	0	0	0	0
DEC-90014	4	3	0	0
DEC-90018	9	9	22	17
DEC-90019	9	9	12	10
DEC-90023	4	4	9	8
DEC-91025	24	20	39	32
DEC-91027	20	14	16	13
Total number	72	61	99	81

#### Set 1

In Set 1, a statistically significantly (p = 0.03) lower frequency of treatments with antibiotics for systemic use (all types within WHO ATC index J01) was found in the delmopinol 0.2% group in comparison with the placebo group (Table 5). The number of treatments with antibiotics was 27% fewer in the delmopinol 0.2% group in comparison with the placebo group. In addition, two separate studies, DEC-90018 and DEC-91025 were both able to show a reduced number of treatments. Thus, in study DEC-90018, the number of treatments was 9 among the 49 patients in the delmopinol 0.2% group vs 22 out of 46 patients in the placebo group, giving a difference of 62% (p < 0.01). In study DEC-91025, the corresponding figures were 24 treatments among the 150 patients in the delmopinol 0.2% group versus 39 out of 150 patients in the placebo group, which was a difference of 38% (p < 0.05).

**Table 5:** Treatments with antibiotics for systemic use (ATC-index group J01), p-value and difference between delmopinol 0.2% and placebo groups in all phase III studies (Set 1)

Calculations based upon number of treatments				
Total no. of treatments	Delmopinol 0.2% 686 patients	Placebo 686 patients	p-value	% difference
	No. of treatments	No. of treatments		
171	72	99	0.0333	27.2
Calculations based upon number of treated patients				
Total no. of treated patients	Delmopinol 0.2% 686 patients	Placebo 686 patients	p-value	% difference
	No. of treated patients	No. of treated patients		
142	61	81	0.0919	24.7

Some patients received more than one treatment with antibiotics. This was more pronounced among the placebo treated patients, and the difference in number of patients in Set 1 treated at least once was not statistically significant (p = 0.09; Table 5). This was also true in the single studies DEC-90018 and DEC-91025 (p = 0.07 and p = 0.09, respectively).

If the calculations in Set 1 were based on antibiotics used for respiratory infections (WHO ATC index groups J01FA and J01HA), the reduction of the number of treatments for delmopinol 0.2% indicated an even greater positive effect of delmopinol 0.2% (Table 6). In this case, the difference was 41% in the delmopinol 0.2% group compared with the placebo group (p = 0.04), and the reduction in the number of treated patients was also statistically significant, 44% (p = 0.04).

**Table 6:** Treatments with antibiotics used for respiratory infections (WHO ATC index groups J01FA and J01HA), p-value and difference between delmopinol 0.2% and placebo groups in all phase III studies (Set 1)

Calculations based upon number of treatments				
Total no. of treatments	Delmopinol 0.2% 686 patients	Placebo 686 patients	p-value	% difference
	No. of treatments	No. of treatments		
67	25	42	0.0443	40.5
Calculations based upon number of treated patients				
Total no. of treated patients	Delmopinol 0.2% 686 patients	Placebo 686 patients	p-value	% difference
	No. of treated patients	No. of treated patients		
56	20	36	0.0398	44.4

#### Set 2

No statistically significant differences in the number of antibiotic treatments were found in the studies where delmopinol 0.1% was included. The percentage difference in these studies was a 19% lower number of treatments in the delmopinol 0.2% group and a 22% lower number in the delmopinol 0.1% group compared to placebo-treated patients. With the exception of study No. DEC-91025, the studies in Set 2 were of a comparably short duration (2–3 months) as seen in Table 1, and – as mentioned above – calculations based on only the long-term (6 months) study DEC-91025 including 450 patients showed statistical significance for delmopinol 0.2% over placebo with respect to treatments (p < 0.05) but not to the number of treated patients (p = 0.09).

#### Set 3

A statistically significant difference between delmopinol and placebo was found, when the three studies in which chlorhexidine was used as a positive control were considered together (Set 3 in Table 7). Two of these studies were 6-month studies and the third was a 5-month study. The number of treatments were 17 for delmopinol 0.2% and 31 for placebo, giving a 46% reduction of treatments for delmopinol 0.2%, which was statistically significant (p = 0.03). However, there was no statistically significant reduction in the number of patients treated with antibiotics in the delmopinol 0.2% group in comparison with the placebo group (Table 7).

**Table 7:** Treatments with antibiotics for systemic use (ATC-index group J01), p-value and difference between delmopinol 0.2% and placebo groups in study Nos. DEC-90014, DEC-90018 and DEC-90023 (Set 3)

Calculations based upon number of treatments				
Total no. of treatments	Delmopinol 0.2% 152 patients	Placebo 150 patients	p-value	% difference
	No. of treatments	No. of treatments		
48	17	31	0.0277	45.9
Calculations based upon number of treated patients				
Total no. of treated patients	Delmopinol 0.2% 152 patients	Placebo 150 patients	p-value	% difference
	No. of treated patients	No. of treated patients		
41	16	25	0.1327	36.8

A comparison between the delmopinol 0.2% and the chlorhexidine groups showed that among the 150 patients in the delmopinol 0.2% group, the number of treatments was 17, in comparison with the 22 treatments among the 152 patients in the chlorhexidine group. The number of treatments was thus 24% lower in the delmopinol 0.2% group, but there was no statistically significant difference between the two treatments (p = 0.39). The same was true for the comparison between chlorhexidine and placebo. Despite a 29% lower number of treatments for chlorhexidine, the statistical calculations did not show any statistically significant difference (p = 0.23).

### Nasal Decongestants (Group R01)

The number of nasal-decongestant treatments and number of patients treated in the delmopinol 0.2% and placebo groups are shown in Table 8.

**Table 8:** Number of treatments with nasal decongestants and the number of patients treated in the delmopinol 0.2% and placebo groups, per study and totally

Study number	Delmopinol 0.2%		Placebo	
	Number of treatments	Number of patients treated	Number of treatments	Number of patients treated
DEC-89016	5	4	3	2
DEC-89017	0	0	0	0
DEC-90014	0	0	0	0
DEC-90018	6	6	12	10
DEC-90019	0	0	0	0
DEC-90023	4	4	12	10
DEC-91025	1	1	3	3
DEC-91027	4	4	13	11
Total number	20	19	43	36

#### Set 1

Table 9 shows that statistically significant lower frequencies of treatments (p = 0.004) with nasal decongestants were found in the delmopinol 0.2% group compared to the placebo group, when all phase III studies (Set 1) were taken together. The number of treatments with nasal decongestants was 53% lower in the delmopinol 0.2% group in comparison with the placebo group. Also the number of patients treated with nasal decongestants was found to be statistically significantly reduced in the delmopinol 0.2% group in comparison with the placebo group (47%, p = 0.03). Two of the studies, DEC-91023 and DEC-91027, both demonstrated a statistically significantly lower number (p < 0.05) of treatments with nasal decongestants for delmopinol 0.2% compared to placebo (data not shown).

**Table 9:** Treatments with nasal decongestants (ATC-index group R01), p-value and difference between delmopinol 0.2% and placebo groups in all phase III studies (Set 1)

Calculations based upon number of treatments				
Total no. of treatments	Delmopinol 0.2% 686 patients	Placebo 686 patients	p-value	% difference
	No. of treatments	No. of treatments		
63	20	43	0.0042	53.4
Calculations based upon number of treated patients				
Total no. of treated patients	Delmopinol 0.2% 686 patients	Placebo 686 patients	p-value	% difference
	No. of treated patients	No. of treated patients		
55	19	36	0.0268	47.2

#### Set 2

When the five studies included in Set 2 were taken together, no statistically significant differences in the number of nasal decongestant treatments were found between delmopinol 0.2% and placebo, despite a lower frequency of as much as 47%. Four of these studies were of short duration, as seen in Table 1. As mentioned above, however, not only the long-term study DEC-91025 showed a statistically significant lower number of treatments compared to placebo, but the short term (3-month) study DEC-91027 did as well.

The number of treatments in the delmopinol 0.2% group showed a statistically significant lower number over the delmopinol 0.1% group (67%, p = 0.01). The placebo group was found to have a lower but non-significant treatment frequency over the delmopinol 0.1% group (data not shown).

#### Set 3

A statistically significant difference (59%, p = 0.01) in the number of treatments with nasal decongestants between delmopinol and placebo was found, when the three studies in which chlorhexidine 0.2% was used as the positive control were considered together (Set 3). However, no statistically significant differences between chlorhexidine 0.2% and placebo (21%, p = 0.51) were found (Table 10). A comparison between the delmopinol 0.2% and the chlorhexidine groups showed that the number of treatments was 48% lower in the delmopinol 0.2% group, but there was no statistically significant difference between the two treatments (p = 0.08).

**Table 10:** Number of nasal decongestant (WHO ATC-index R01) treatment periods of patients in study Nos. DEC-90014, DEC-90018 and DEC-90023 (Set 3)

Calculations based upon number of treatments					
Total no. of treatments	Delmopinol 0.2% 152 patients	Chlorhexidine 150 patients	Placebo 150 patients	p-value	% difference
34	10	-	24	0.0108	58.9
43	-	19	24	0.5102	20.8
29	10	19	-	0.0811	48.1

The number of treated patients was the same as the corresponding numbers of treatments in the delmopinol 0.2% and the placebo groups. The same figures were thus obtained in these two groups with respect to the difference in the numbers of treated patients (59%, p = 0.01). The corresponding calculations with respect to patients could not be performed for the chlorhexidine group, due to missing data.

### Other Agents

The compounds used in group M01 (anti-inflammatory/anti-rheumatic non-steroids) were dominated by ibuprofen or naproxen, and in group N02B (other analgesics and antipyretics), mostly acetylsalicylic acid or paracetamol were used. As the M01 and N02B pharmaceuticals are used mainly to treat pain of mild or moderate intensity, the groups were pooled. A slightly lower (7%), non-significant frequency (p = 0.45) of treatments was found in the delmopinol 0.2% group. No other reductions in frequencies of concomitant treatment in these studies with delmopinol were found.

## Discussion

The main purpose of the clinical phase III studies was to study the effects and side-effects of Decapinol Mouthwash 2 mg/ml (delmopinol 0.2%) on patients with periodontal diseases. On three or four occasions during the studies, the patients were examined by the investigators to determine efficacy and adverse events. With respect to efficacy, the studies showed that rinsing with delmopinol 0.2% fulfilled the ADA effectiveness criteria for controlling plaque and gingivitis, and was safe to use. A transient numbing sensation and taste disturbances were reported as the most common adverse effects of delmopinol.^1^ However, the side-effects seemed to be well tolerated, since they did not cause an increased frequency in withdrawal rate. As shown in Table 1, that rate was close to the placebo group and much lower than for the chlorhexidine 0.2% solution. An explanation for the adverse effects of delmopinol – as well as for its pharmacokinetic behaviour after rinsing – was suggested in a study on the influence of delmopinol on the oral mucosa, where it was shown that delmopinol was rapidly adsorbed and retained for up to 4 h in this tissue.^34^ However, delmopinol’s adsorption to and absorption by the oral mucosa have also been suggested to have a positive side-effect: a statistically significant and clinically important reduction of the frequency in occurrence of aphthous stomatitis was found.^36^

This positive finding made it interesting to analyse whether delmopinol treatment also had an effect on the use of concomitant medication in the studies. Statistically significant differences in the number of treatments of concomitant medication were found for antibiotics for systemic use (ATC index group J01), and for nasal preparations (ATC index group R01). With respect to antibiotics, a statistically significant reduction of 27% (Set 1) to 45% (Set 3) in the number of treatments was obtained for antibiotics in the delmopinol 0.2% group in comparison with placebo. Furthermore, it should be noted that statistical significance was found not only in the total number of studies added, but also in single studies (DEC-90018 and DEC-91025). Therefore, a false positive outcome seems unlikely. Most interestingly, we found that delmopinol seemed to have a greater positive effect on infections in the respiratory tract, since the reduction in frequency of antibiotics for respiratory infections was more pronounced than for antibiotics in general. Thus, in Set 1, a statistically significant reduction of 27% in the number of treatments was obtained for antibiotics (all indications) in the delmopinol 0.2% group in comparison with placebo, but a 41% reduction of the number of treatments with antibiotics used for respiratory infections. This corroborates with a number of authors who pointed out that the oral microbiome has easier access to the respiratory system compared to other organ systems, due to the close connection between the oral cavity and the upper respiratory tract.^2,20,24^

However, no statistically significant differences in the number of antibiotic treatments were found in the studies where delmopinol 0.1% solution was used (Set 2). Both delmopinol 0.2% and delmopinol 0.1% were about 20% more effective than placebo, but this was not statistically significant. A possible explanation might be that – with the exception of study No. DEC-91025 (which did show a significant difference) – those studies were of shorter duration, only 2-3 months in comparison with 5-6 months, as seen in Table 1.

The positive effect of delmopinol 0.2% over placebo was also obtained regarding the use of nasal decongestants, compounds that are frequently used in connection with the common cold and influenza.^8^ The obtained percentage differences between the two groups are high, about 55%, and statistically significant (Set 1 and Set 3). In Set 2, no statistically significant effect was obtained in terms of reducing nasal-decongestant use, despite a reduction in treatment numbers of 47%. However, both study DEC-91025 as well as study DEC-91027 in Set 2 showed a statistically significant lower number (p < 0.05) of treatments with nasal decongestants compared to placebo, and there was a statistically significantly better effect of delmopinol 0.2% over delmopinol 0.1%.

The subgroup where chlorhexidine 0.2% was the active control (Set 3) included two 6-month studies and one 5-month study. Statistically significantly lower number of treatments with antibiotics as well as nasal decongestants were found in the delmopinol 0.2% group vs the placebo when these studies were analysed together, while no statistically significant differences were detected between chlorhexidine and placebo. A comparison between delmopinol 0.2% and chlorhexidine 0.2% did not show any statistically significant differences in the number of treatments with antibiotics or nasal decongestants. Despite a 24% or 48% lower number of treatments with antibiotics and nasal decongestants in the delmopinol group over the chlorhexidine group, respectively, no statistically significant differences were found.

Delmopinol thus seems to be at least as good as chlorhexidine with respect to reducing concomitant treatment of antibiotics and nasal decongestants. The two compounds have quite different mechanisms of action. Chlorhexidine possesses broad-spectrum antimicrobial activity that is attributed to disruption of the cellular plasma membrane.^25^ The surface-active compound delmopinol, however, is much less antimicrobial, but changes the surface properties of bacteria, teeth and oral mucosa.^11,31,34^ One important aspect of this is the interference of delmopinol with bacterial extracellular enzymes responsible for the synthesis of polysaccharides necessary for plaque growth and stability.^26,28,38^ This will affect plaque maturation, and in short-term clinical studies, delmopinol has been shown to maintain the early streptococci-dominated bacterial plaque composition, which is considered to be related to a healthy gingiva, thereby preventing or delaying colonisation by rods, filaments and curved rods.^6,7,27^ A possible explanation for the reduction of antibiotics and nasal decongestants in patients rinsing with delmopinol 0.2%, is thus that the compound contributes to the maintenance of a “normal” (non-pathogenic) oral bacterial flora, and thereby prevents pathogenic bacteria from invading the respiratory tract.

The statement that delmopinol has a much lower antimicrobial profile than chlorhexidine is based upon their minimum inhibitory concentrations (MIC). However, since the biofilm is an effective survival structure that protects the resident organisms from exogenous, potentially harmful factors, the MIC value is often not predictive of clinical efficacy.^4,23,37^ Instead, testing agents for their effect on bacterial biofilms is a more reliable method for selection of clinically efficacy. The ability for chlorhexidine to penetrate dental plaque has been described as limited.^19^ This was also supported in an in-vitro study of the antimicrobial effects of delmopinol and some antimicrobial compounds on Gram-positive and Gram-negative bacteria, where it was shown that the difference in the MICs of chlorhexidine for planktonic and biofilm bacteria were quite large (the ratio varied between 8 and 63), whereas the corresponding ratio for delmopinol was much lower, 1 to 2.33. It was suggested that the reason for the low penetration in dental plaque of the cationic compound chlorhexidine was due to binding to negative groups in the extracellular matrix of polymeric substances (EPS) surrounding the biofilm bacteria.^33^ Since chlorhexidine has a pKa-value of 10.8 (http://www.pubchem.ncbi.nlm.nih.gov>compound), it will always be cationic in the oral cavity. For delmopinol, however, having a pKa of 7.1, there are equimolar concentrations of cationic and non-ionic delmopinol at neutral pH, and the presence of a major fraction of the non-ionic form of delmopinol was shown to be of great importance for its antimicrobial effects on sessile bacteria. Thus, when delmopinol was compared with its cationic analogue “quaternary delmopinol” with respect to their MICs for planktonic and biofilm bacteria, similar figures were obtained for quaternary delmopinol as for delmopinol with respect to planktonic bacteria, but much higher concentrations of quaternary delmopinol were needed to kill the biofilm bacteria.^33^

The presence of both non-ionic and cationic delmopinol is important also for its adsorption to and absorption by the oral mucosa, that together with salivary associated films constitute a protective barrier along with the desquamation process. It has been shown that delmopinol, administered by rinsing the mouth for 1 min with an unbuffered aqueous solution of delmopinol 0.2%, was rapidly adsorbed and retained in the oral mucosa and then slowly released from this tissue into the systemic circulation.^13,34^ It was proposed that the major part of adsorbed delmopinol consists of its non-ionized form, which can be explained by the pKa-value for delmopinol, the great difference in water solubility between its cationic and non-ionized forms, the pH of the oral cavity, and the concentration of delmopinol in the administered aqueous solution.^34^ This retention of delmopinol to the oral mucosa should also be taken into consideration as a possible explanation for the reduction of antibiotics and nasal decongestants after rinsing with delmopinol 0.2%.

This examination of clinical data with respect to the use of concomitant medication shows that a mouthwash of delmopinol 0.2%, when used twice daily with a rinse time of 1 min, in addition to the patient’s normal oral hygiene procedures, reduces the need for antibiotics and nasal decongestants. Of particular interest is the reduction of antibiotics, since many governments together with the World Health Organization (WHO) and the United Nations (UN) have joined efforts for a restrictive use of antibiotics in order to reduce resistance development. The more antibiotics that are used, the more the antimicrobial resistance will increase, which in turn is associated with an elevated risk of treatment failure and relapsing infections, resulting in growing healthcare costs. Preventive work with hygiene in society will reduce the number of infections, which in turn reduce the need for antibiotics.

It should be kept in mind that the demonstrated effects of delmopinol on concomitant medication were obtained using a formulation and handling instructions that were developed for patients with periodontal disease. Additional clinical studies, designed to further evaluate to what extent treatment with delmopinol 0.2% may decrease the need for antibiotics and nasal decongestants, should be performed in order to improve oral hygiene among patients who are at risk (e.g., patients with cystic fibrosis, chronic obstructive pulmonary disease, elderly living in long-term care institutions). Furthermore, it is possible that the positive effect of delmopinol on concomitant medication can be further improved, since each therapy requires distinct handling procedures and formulations in order to optimise treatment and minimise side-effects.^3^

## Conclusion

Administered to patients with gingivitis, an unbuffered mouthwash solution containing delmopinol 0.2% (along with their normal oral hygiene procedures) has been shown to statistically significantly reduce consumption of concomitant medication, specifically, antibiotics and nasal decongestants. These results should be followed up with clinical studies on patients who are at risk and those living in long-term care institutions.

## References

[ref1] Addy M, Moran J, Newcombe R (2007). Meta-analyses of studies of 0.2% delmopinol mouth rinse as an adjunct to gingival health and plaque control measures. J Clin Periodontol.

[ref2] Azarpazhooh A, Leake J (2006). Systematic review of the association between respiratory disease and oral health. J Periodontol.

[ref3] Bartlett J, van der Voort Maarschalk K (2012). Understanding the oral mucosal absorption and resulting clinical pharmacokinetics of asenapine. AAPS PharmSciTech.

[ref4] Ceri H, Olson M, Stremick C, Read R, Morck D, Buret A (1999). The Calgary biofilm device: new technology for rapid determination of antibiotic susceptibility of bacterial biofilms. J Clin Microbiol.

[ref5] Claydon N, Hunter L, Moran J, Wade W, Kelty E, Movert R (1996). A 6-month home-usage trial of 0.1% and 0.2% delmopinol mouthwashes (I). Effects on plaque, gingivitis, supragingival calculus and tooth staining. J Clin Periodontol.

[ref6] Collaert B, Attström R, de Bruyn H, Movert R (1992). The effect of delmopinol rinsing on dental plaque formation and gingivitis healing. J Clin Periodontol.

[ref7] Collaert B, Edwardsson S, Attström R, Hase J, Åström M, Movert R (1992). Rinsing with delmopinol 0.2% and chlorhexidine 0.2%: short-term effect on salivary microbiology, plaque, and gingivitis. J Periodontol.

[ref8] Deckx L, De Sutter AIM, Guo L, Mir NA, van Driel ML (2016). Nasal decongestants in monotherapy for the common cold (Review). Cochrane Database of Systematic Reviews.

[ref9] DeRiso A, Ladowski J, Dillon T, Justice J, Peterson A (1996). Chlorhexidine gluconate 0.12 % oral rinse reduces the incidence of total nosocomial respiratory infection and nonprophylactic systemic antiobiotic use in patients undergoing heart surgery. Chest.

[ref10] Elworthy AJ, Edgar R, Moran J, Addy M, Movert R, Kelty E (1995). A 6-month home-usage trial of 0.1% and 0.2% delmopinol mouthwashes (II). Effects on the plaque microflora. J Clin Periodontol.

[ref11] Elworthy AJ, Wade WG (1995). Antimicrobial properties of delmopinol against oral bacteria. Lett Appl Microbiol.

[ref12] Eriksson B, Hallström G, Ottersgård Brorsson AK, Svensson L, Gunnarsson PO (2000). Metabolic fate of delmopinol in man after mouth rinsing and after oral administration. Xenobiotica.

[ref13] Eriksson B, Ottersgård Brorsson A-K, Hallström G, Sjödin T, Gunnarsson PO (1998). Pharmacokinetics of 14C-delmopinol in the healthy male volunteer. Xenobiotica.

[ref14] Fischer R G, Lira Junior R, Retamal-Valdes B, de Figueiredo L C, Malheiros Z, Stewart B (2020). Periodontal disease and its impact on general health in Latin America. Section V: Treatment of periodontitis. Braz Oral Res.

[ref15] Hase J, Attström R, Edwardsson S, Kelty E, Kisch J (1998). 6-month use of 0.2% delmopinol hydrochloride in comparison with chlorhexidine digluconate and placebo. (I). Effect on plaque formation and gingivitis. J Clin Periodontol.

[ref16] Hase J, Edwardsson S, Rundegren J, Attström R, Kelty E (1998). 6-month use of 0.2% delmopinol hydrochloride in comparison with 0.2% chlorhexidine digluconate and placebo (II). Effect on plaque and salivary microflora. J Clin Periodontol.

[ref17] Klinge B, Matsson L, Attström R, Edwardsson S, Sjödin T (1996). Effect of local application of delmopinol hydrochloride on developing and early established supragingival plaque in humans. J Clin Periodontol.

[ref18] Lang N, Hase J, Grassi M, Hämmerle CHF, Weigel C, Kelty E (1998). Plaque formation and gingivitis after supervised mouthrinsing with 0.2% delmopinol hydrochloride, 0.2% chlorhexidine digluconate and placebo for 6 months. Oral Dis.

[ref19] Marsh P, Lewis M, Rogers H, Williams D, Wilson M (2016). Marsh & Martin’s Oral Microbiology.

[ref20] Mojon P (2002). Oral health and respiratory infection. J Can Dent Assoc.

[ref21] Moran J, Addy M, Wade WG, Maynard JH, Roberts SE, Åström M (1992). A comparison of delmopinol and chlorhexidine on plaque regrowth over a 4-day period and salivary bacterial counts. J Clin Periodontol.

[ref22] Neilands J, Troedsson U, Sjödin T, Davies JR (2014). The effect of delmopinol and fluoride on acid adaptation and acid production in dental plaque biofilms. Arch Oral Biol.

[ref23] Olson M, Ceri H, Morck D, Buret A, Read R (2002). Biofilm bacteria: formation and comparative susceptibility to antibiotics. Can J Vet Res.

[ref24] Paju S, Scannapieco FA (2007). Oral biofilms, periodontitis, and pulmonaty infections. Oral Dis.

[ref25] Poppolo Deus F, Ouanounou A (2022). Chlorhexidine in dentistry. Pharmacology, uses, and adverse effects. Int Dent J.

[ref26] Rundegren J, Arnebrant T (1992). Effect of delmopinol on the viscosity of extracellular glucans produced by Streptococcus mutans. Caries Res.

[ref27] Rundegren J, Bondesson Hvid E, Johansson M, Åström M (1992). Effect of 4 days mouth rinsing with delmopinol or chlorhexidine on the vitality of plaque bacteria. J Clin Periodontol.

[ref28] Rundegren J, Simonsson T, Petersson L, Hansson E (1992). Effect of delmopinol on the cohesion of glucan-containing plaque formed by Streptococcus mutans in a flow cell system. J Dent Res.

[ref29] Rundegren J, Sjödin T, Petersson L, Hansson E, Jonsson I (1995). Delmopinol interactions with cell walls of gram-negative and gram-positive oral bacteria. Oral Microbiol Immunol.

[ref30] Sabharwal A, Stellrecht E, Scannapieco F (2021). Associations between dental caries and systemic diseases: a scoping review. BMC Oral Health.

[ref31] Simonsson T, Arnebrant T, Petersson L, Hvid EB (1991). Influence of delmopinol on bacterial zeta-potentials and on the colloidal stability of bacterial suspensions. Acta Odontol Scand.

[ref32] Simonsson T, Hvid EB, Rundegren J, Edwardsson S (1991). Effect of delmopinol on in vitro dental plaque formation, bacterial acid production and the number of microorganisms in human saliva. Oral Microbiol Immunol.

[ref33] Sjödin T (2019). The pH-dependent effect of cationic and non-ionic delmopinol on planktonic and biofilm bacteria. Arch Oral Biol.

[ref34] Sjödin T, Diogo Löfgren C, Glantz PO, Christersson C (2020). Delmopinol – adsorption to and absorption through the oral mucosa. Acta Odontol Scand.

[ref35] Sjödin T, Håkansson J, Sparre B, Ekman I, Åström M (2011). Pharmacokinetics and clinical efficacy of delmopinol in an open rinse time study in healthy volunteers. Am J Dent.

[ref36] Sjödin T, Movert R, Åström M (2022). The effect of delmopinol mouthwash on aphthous stomatitis. J Dental Health Oral Res.

[ref37] Socransky S, Haffajee A (2002). Dental biofilms: difficult therapeutic targets. Periodontology 2000.

[ref38] Steinberg D, Beeman D, Bowman W (1992). The effect of delmopinol on glucosyltransferase adsorbed on to saliva-coated hydroxyapatite. Arch Oral Biol.

[ref39] Vassilakos N, Arnebrant T, Rundegren J (1993). In vitro interactions of delmopinol hydrochloride with salivary films adsorbed at solid/liquid interfaces. Caries Res.

